# Growth and Adult Height during Human Growth Hormone Treatment in Chinese Children with Multiple Pituitary Hormone Deficiency Caused by Pituitary Stalk Interruption Syndrome: A Single Centre Study

**DOI:** 10.4274/jcrpe.galenos.2019.2019.0086

**Published:** 2020-03-19

**Authors:** Fengxue Wang, Jinyan Han, Zengmin Wang, Xiaohong Shang, Guimei Li

**Affiliations:** 1 Shandong Provincial Hospital affiliated to Shandong University, Department of Pediatrics, Shandong, China

**Keywords:** Pituitary stalk interruption syndrome, growth velocity, human growth hormone treatment, adult height

## Abstract

**Objective:**

The aim was to assess growth velocity (GV) during human recombinant growth hormone (hGH) treatment of children with multiple pituitary hormone deficiency (MPHD) caused by pituitary stalk interruption syndrome (PSIS) and to analyze the characteristics of patients that attained normal adult heights.

**Methods:**

Data from 74 (16 female) children with MPHD caused by PSIS with GH, thyroid stimulating hormone, gonadotropin and adrenocorticotropic hormone deficiencies were collected. Subjects were divided into groups: 12 pre-pubescent females (Female-Group) and 36 pre-pubescent males (Male-Group 1). The remaining 22 males were further sub-divided into two groups (Male-Group 2 and Male-Group 3) according to the initiation of gonadotropin replacement treatment, based on bone age and height.

**Results:**

No differences in change in height standard deviation score (△HtSDS) and GV were observed at different time points of hGH treatment between the Female-Group and Male-Group 1 (p>0.05). GV was significantly greater in the first year of hGH therapy than in subsequent years: Female-Group p=0.011; Male-Group 1 p<0.001; Male-Group 2 p=0.005; and Male-Group 3 p=0.046. Adult height was achieved by 23 (19 males and 4 females) patients. The total gain in height positively correlated with the GV during the first year (r=0.626, p<0.001).

**Conclusion:**

GV during hGH treatment were similar amongst pre-pubescent males and females with MPHD caused by PSIS. GV during the first year of hGH treatment appears to be an effective predictor of final height in patients with MPHD caused by PSIS.

**What is already known on this topic?**

Pituitary stalk interruption syndrome (PSIS) is a kind of congenital disease associated with multiple pituitary hormone deficiency (MPHD). Human recombinant growth hormone (hGH) treatment is the optimal therapy for short stature in children with isolated GH deficiency and can effectively increase growth velocity (GV) to attain adult heights within the target range.

**What this study adds?**

GVs during hGH treatment were similar amongst pre-pubescent males and females with MPHD caused by PSIS. The GV during the first year of hGH treatment is an effective predictor of future height outcomes in patients with MPHD caused by PSIS.

## Introduction

Pituitary stalk interruption syndrome (PSIS) is characterized by the occurrence of a thin or absent pituitary stalk, hypoplasia of adenohypophysis, and ectopic neurohypophysis on magnetic resonance imaging (MRI) of the hypothalamo-pituitary region (1). It is a rare congenital disease associated with multiple pituitary hormone deficiencies (MPHD). MPHD, by definition, represents an impaired production of one or more anterior pituitary hormones in addition to growth hormone (GH) and is a chronic, lifelong condition (2,3,4,5).

PSIS as the most common cause of MPHD, was first reported by Fujisawa et al (6) in 1987. Children with PSIS typically present with growth reduction and delayed puberty, leading to significant distress to children and their families. Recombinant human GH (hGH) treatment is the optimal therapy for short stature in children with isolated GH deficiency (IGHD) and can effectively increase height velocity to attain adult heights within the target range (7,8). Whether the benefits of hGH treatment in patients with MPHD caused by PSIS are consistent with those observed in IGHD is currently unknown.

In this study, we assessed the growth velocity (GV) of patients with MPHD caused by PSIS who were administered hGH. We further analyzed the characteristics of patients that subsequently attained adult heights.

## Methods

### Patients and Grouping

In this retrospective study, data from patients diagnosed with PSIS during childhood and adolescence presenting during the time period from January 2008 to November 2018 in pediatric endocrine outpatients of Shandong Provincial Hospital were analyzed. Patients with confirmed GH, thyroid stimulating hormone (TSH), gonadotropin and adrenocorticotropic hormone (ACTH) deficiencies were included in the analysis. None of the patients had spontaneous puberty and puberty was induced *via* gonadotropin or sex hormone treatment. Pre-pubescent subjects at presentation were divided into the Female-Group and Male-Group 1. Additionally, patients were treated with hGH followed by gonadotropin, administered at the appropriate age and the male patients from this subgroup formed Male-Group 2. The final study group consisted of male patients who received initial hGH and gonadotropin treatment simultaneously and these were designated Male-Group 3.

### Ethics

The study was approved by the Medical Ethics Committee of Shandong Provincial Hospital, affiliated to Shandong University (approval number: 2019-053). Patients or their parents/guardians provided verbal consent for anonymized data to be collated and analyzed, approved by the ethics committee.

### Diagnostic Criteria

The diagnosis of PSIS was based on cranial MRI of the hypothalamus and pituitary gland. Imaging criteria for a diagnosis of PSIS included an absent or thin pituitary stalk, hypoplasia of the anterior pituitary gland, and ectopic location of the posterior pituitary. MRI scans were performed using a 3.0 T Scanner (Siemens, Erlangen, Germany) in the sagittal and coronal planes on T1 and T2 weighted imaging (slice thickness 3 millimeters).

Bone age (BA) was determined by left hand and wrist X-ray images according to the method of Greulich and Pyle. Hypophyseal hormone levels were investigated in each patient. The pituitary axis was examined using the following tests: (1) GH deficiency (GHD) was diagnosed in the absence of a significant peak in GH secretion after more than one stimulation test. Diagnosis was based on GH peak concentrations <10 ng/mL, following two independent GH provocation tests which were either an intravenous arginine test, 0.5 mg/kg; maximum dosage 30 mg and/or an oral levodopa test, 10 mg/kg; maximum dosage: 500 mg [arginine produced by Harbin Pharmaceutical Group Co., Ltd. in Harbin, Heilongjiang, China and levodopa produced by Aikanglide Pharmaceutical (Zhejiang Co., Ltd in Quzhou, Zhejiang, China). Patients received GH provocation tests on the basis of normal thyroid and adrenal functions (2). TSH deficiency was defined as a low serum free thyroxine (fT4) of <12.0 pmol/L (reference range: 12.0-22.0 pmol/L) with concomitantly normal or decreased serum TSH (reference range: 0.27-4.2 uIU/mL) (3). ACTH deficiency was assessed by either decreased serum cortisol (COR) levels in the morning (COR <138 nmol/L) or an impaired serum COR concentration increase (COR <550 nmol/L) during insulin-induced hypoglycemia with inappropriately low serum ACTH concentrations (4). Gonadotropin deficiency was based on the gonadotropin hormone-releasing hormone-stimulation test [triptorelin: 2.5 µg/kg administered as a subcutaneous injection at a maximum dosage of 100 µg with cut-off points for a blunted response of 2.8 mIU/mL for luteinizing hormone (LH) and/or 3.7 mIU/mL for follicle-stimulating hormone (FSH)]; or basal levels of FSH and LH below the sensitivity range of the assay (<0.1 mIU/mL) on the basis of delayed or absent pubertal development (9,10). Serum GH levels were measured using chemiluminescence assays (Cobas E170, Roche Diagnostics, Germany). Serum fT4, TSH, ACTH, COR, FSH and LH were measured using chemiluminescence assays (Siemens Healthcare Diagnostics, USA). All patients underwent the same testing protocol and all tests were performed after overnight fasting (5).

### Height and Weight

Height was measured in centimeters (cm) in the morning by the same medical team. Height measurements were standardized for age and sex and expressed as standard deviation scores (HtSDS) relative to chronological age (CA) according to Growth Charts for Chinese Children and Adolescents (2009) (5,11). The GV during hGH treatment was analyzed each year and was calculated in cm through the difference in previously recorded height. △HtSDS was calculated by the difference from the previously recorded HtSDS. The parental target height was calculated according to the following formula: ([height of the father + height of the mother] / 2) - 6.5 cm for girls and +6.5 cm for boys, and also expressed as standard deviation scores (SDS) according to the Growth Charts for Chinese Children and Adolescents (2009) (11).

Weight was measured in kilogrammes (kg) in the morning, in the fasting state with no shoes and light clothes at each visit. Body mass index (BMI) was calculated using the standard formula of weight (kg) divided by height squared (meters). BMI values were transformed into BMI-SDS, based on Normative Values for Chinese Children and Adolescents (2009) (5,11) to adjust for the confounding effects of age and sex.

### Treatment and Follow-up

Patients received hormone replacement therapy according to their known hormone deficiencies. Hydrocortisone and L-thyroxine were immediately administered once ACTH and TSH deficiency were confirmed. The dosages of hydrocortisone were between 10-15 mg/m^2^/day, oral administration, divided into two daily doses. The dosages of L-thyroxine were between 1.5-2.0 ug/kg/day, oral administration, qd. The dosages of hydrocortisone and L-thyroxine were adjusted to maintain the levels of fT4, COR, blood glucose and serum electrolytes within normal ranges. In patients with normal thyroid and adrenal hormone levels (including patients who were corrected with hydrocortisone and L-thyroxine) hGH was administered (hGH produced by ChangChun GeneScience Pharmaceuticals Co., Ltd in Changchun, Jilin, China). The dosage of hGH was between 0.10-0.15 IU/kg/day) and administered by daily injection 7 days/week. Puberty was initiated in 26 patients through the administration of gonadotropin; exogenous human chorionic gonadotropin (hCG) and/or urine-derived human menopause gonadotropin (hMG) therapy (hCG and hMG from Livzon Pharmaceutical Group Inc., in Zhuhai, Guangdong, China). For male patients the dosage of intramuscular (i.m.) hCG was 2000 IU, twice per week, whilst for female patients 75 IU i.m. hMG was administered twice a week. Boys had a pretreatment phase of hCG for three months. As serum testosterone (TO) reached normal values, hCG combined with hMG was administered to improve sexual development. If the TO concentration did not attain normal values, exogenous TO (TO undecanoate Catalent France Beinheim S.A. in France: 80-160 mg/day, oral administration) was given to induce puberty (12).

All patients were followed in outpatient clinic at three monthly intervals. Compliance with treatment was assessed at each visit, and patients underwent complete physical examinations by the same medical team, including height and weight measurements during and after treatment. Biochemical and hormone status were also assessed.

### Statistical Analysis

Data were analyzed using Statistical Package for the Social Sciences software (IBM SPSS for Windows, Version 25; IBM Corp., Armonk, NY, USA). The continuous data in our research were tested for normal distribution using Kolmogorov-Smirnov test, and found to be approximately normally distributed. The descriptive statistics of the quantitative variables were presented as means ± standard deviations (SD). Groups were compared using the Student’s t-test. Pearson’s correlation was used to assess the relationships between various parameters. The threshold for statistical significance was <0.05.

## Results

In total, data from 74 patients (58 males and 16 females) was analyzed. Amongst these patients, 48 (36 males and 12 females) who received hGH treatment and other deficient hormones, excluding gonadotropin during the study period, were pre-pubescent. Pre-pubescent subjects at presentation were divided into the Female-Group (n=12) and Male-Group 1 (n=36). Additionally, 13 patients (10 males and 3 females) were treated with hGH followed by gonadotropin which was administered at the appropriate age to induce puberty. The 10 male patients in this latter group formed Male-Group 2. Lastly, 13 subjects (12 males and 1 female) received combined hGH and gonadotropin treatment at presentation. These 12 males were grouped into Male-Group 3.

### Growth Velocity of Pre-pubertal Patients (Female-Group and Male-Group 1) Treated with Human Growth Hormone Alone

**Figure 1:**
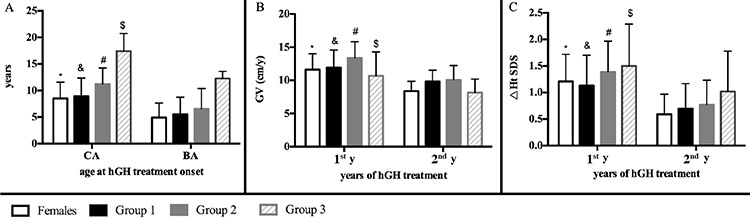
**A, B, C)** The age, growth velocity (GV) and △HtSDS at different points during hGH treatment. A) CA at the hGH treatment onset of four groups was significantly larger than their BA. *p=0.006, ^&^p≤0.001, ^#^p=0.009, ^$^p≤0.001. B) GV in the first year during hGH treatment was significantly higher than in the second year. *p=0.011, ^&^p≤0.001,^ #^p=0.005, ^$^p=0.046. C) The difference in height SDS between the first year and the hGH treatment onset was higher than in the second year for Female-Group, Male-Group 1 and Male-Group 2. *p≤0.001, ^&^p=0.003, ^#^p=0.013, ^$^p=0.144 GV: growth velocity, △HtSDS: height standard deviation score, hGH: human growth hormone, CA: chronological age, BA: bone age

CA, BA, BMI-SDS and HtSDS during hGH treatment were 8.51±3.08 years, 4.91±2.75 years, -0.20±1.11 and -3.60±1.76 in the Female-Group and 8.94±3.42 years, 5.51±3.22 years, 0.20±1.35 and -2.97±1.23 in the Male-Group 1. There were no differences in CA and BA, BMI-SDS and HtSDS at hGH treatments between pre-pubescent females (Female-Group) and males (Male-Group 1) (p=0.698, p=0.653, p=0.358 and p=0.175). The CA was significantly larger than the BA in both groups (p=0.006 for Female-Group and p<0.001 for Male-Group 1) (Figure 1A).

There were no differences in HtSDS at any point during hGH treatment (p=0.292 and p=0.157). For Female-Group patients, the GV was 11.63±2.38 cm/y in the first year and 9.37±1.48 cm/y in the second year. The GV of Male-Group 1 in the first year was 11.95±2.62 cm/y compared to 9.83±1.71 cm/y in the second year. No differences in GV at any time point during hGH treatment between pre-pubescent females (Female-Group) and males (Male-Group 1) (p=0.710 for the first year and p=0.410 for the second year) were observed. GV in the first year was higher than in the second year for both two groups (p=0.011 for the Female-Group and p<0.001 for the Male-Group 1) (Figure 1B). The △HtSDS1 for the Female- and Male-Group 1 were significantly higher than the △HtSDS2 values (1.21±0.51 vs 0.59±0.38 and p≤0.001 for Female-Group, 1.13±0.57 vs 0.70±0.47, p=0.003 for Male-Group 1, respectively) (Figure 1C). Detailed information is shown in Table 1.

### Growth Velocity Male-Group 2 and Male-Group 3 Treated with hGH and Gonadotropin

Detailed information of CA, BA, height SDS and GV during hGH or gonadotropin treatment are shown in Tables 1 and 2. The CA following hGH treatment of the Male-Group 2 was larger than BA (11.24±2.99 years vs 6.55±3.82 years, p=0.009). No differences in CA and BA at the initiation of hGH treatment between Male-Group 1 and Male-Group 2 were observed (p=0.060 and p=0.390, respectively). CA at the initiation of hGH treatment (hGH + gonadotropin treatment) of Male-Group 3 was larger than BA (17.42±3.32 years vs 12.25±1.37 years, p≤0.001). The CA of Male-Group 3 was significantly higher than that of Male-Group 1 and Male-Group 2 (both p≤0.001) which was also observed for BA values (both p≤0.001).

The GV during the first two years of hGH treatment in the Male-Group 2 were 13.37±2.45 and 10.08±2.16 cm/year. GV during the first two years of hGH (hGH + gonadotropin) treatment of Male-Group 3 were 10.68±3.59 and 8.16±2.03 cm/year. We observed no differences in GV during the first year of hGH treatment amongst the three groups (p=0.132 between Group 1 and Group 2, p=0.193 between Group 1 and Group 3, and p=0.058 between Group 2 and Group 3). During the second year, no differences in GV were observed between Groups 1 and 2 (p=0.701), but the GV of Group 3 was significantly lower than the other two male groups (p=0.007 between Male-Group 1 and Male-Group 3, and p=0.044 between Male-Group 2 and Male-Group 3).

GV during the first year of hGH treatment was higher than the second year for the three groups (p≤0.001 for Male-Group 1, p=0.005 for Male-Group 2 and p=0.046 for Male-Group 3, respectively) (Figure 1B). The differences in △HtSDS between the first and second year of hGH treatment for Male-Group 2 were also statistically significant (1.39±0.58 vs 0.77±0.41, p=0.013). However, differences in the △HtSDS during the first two years of hGH treatment did not change in the Male-Group 3 (1.50±0.79 vs 1.02±0.76, p=0.144) (Figure 1C).

The CA during the initiation of hGH + gonadotropin treatment of Male-Group 2 (13.39±2.80) were significantly lower than those of Male-Group 3 (p=0.007), but no differences in BA at the initiation of hGH + gonadotropin treatment between the groups were observed (p=0.066). The GV in the first year of hGH + gonadotropin treatment in Group 3 was significantly higher than that of Group 2 (p=0.041), which was similar for △HtSDS (p=0.004).

### Characteristics of Patients Achieving Adult Height

In total, 23 patients (19 males) with PSIS reached adult height following hGH treatment. For male patients, 18/19 attained adult height >-2 SD, yet only seven reached a height above the 50^th^ percentile (adult height ≥172.7 cm or adult HtSDS ≥0). For females, all patients (4/4) reached a normal adult height range and all were above the 50^th^ percentile (adult height ≥160.6 cm or adult HtSDS >0).

The mean adult height was 168.5±6.1 cm (HtSDS=-0.47±1.11) for males and 164.0±2.9 cm (HtSDS=0.77±0.49) for females. The parental target height was 170.1±4.9 cm (HtSDS=-0.43±0.82) for males and 160.8±1.3 cm (HtSDS=0.26±0.03) for females. The mean age at initiation of hGH treatment in females was 10.4±0.8 years and 14.4±3.5 years in males. The mean BA at initiation of hGH treatment in females was 8.4±0.8 years and 9.9±3.5 years for males. Mean HtSDS at hGH treatment onset was -3.11±1.86 for males and -1.75±0.23 for females, respectively. The mean GV in the first year of hGH treatment were 11.0±3.2 cm and 12.9±1.9 cm for males and females, respectively. The mean total height gain was 23.9±15.6 cm and 20.9±4.9 cm for males and females, respectively.

**Figure 2:**
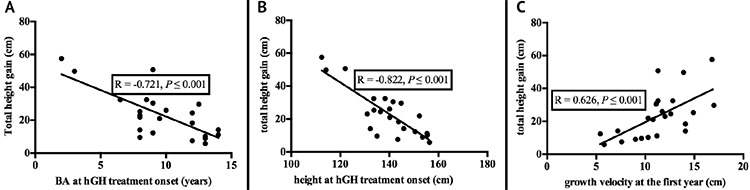
**A, B, C)** Correlation between total height gain and bone age, height and growth velocity at first year hGH: human growth hormone, BA: bone age

A negative correlation was found between the total height gain and BA at hGH onset and between total height gain and height at hGH treatment onset (r=-0.721, p<0.001; and r=-0.822, p<0.001, respectively). Moreover, a positive correlation was observed between total height gain and GV in the first year of hGH treatment (r=0.626, p<0.001). Figure 2 graphically depicts these correlations.

## Discussion

Various anterior pituitary hormone deficiencies and clinical presentations are common in PSIS patients. To date, studies on the growth of children and adolescents with PSIS during the course of hGH treatment are sparse and continuous follow-up to adult age is rarely reported (5). In this retrospective study, measurements were performed in 74 patients with PSIS. Our analysis included long-term patient follow-up, performed at regular short intervals, by the same team of healthcare professionals. Despite certain limitations, the results provided various noteworthy observations. It has been reported that the addition of gonadotropins may affect GV in pubertal children (4), so male PSIS patients were divided into three groups on the basis of their gonadotropin treatment protocols.

All children short in stature can receive hGH treatment to decrease linear height deficits. The CA of PSIS patients receiving hGH treatment in this study were older than those of previous studies (13). Chinese parents are familiar with the idea of “delayed puberty”, leading to delay in referral and older age at presentation in Chinese patients. A large number of children were from rural areas with an undeveloped economy, in which the attention to growth and development is low. These factors contribute to the older age of PSIS children and adolescents in our cohort. The BA of all the PSIS children in our study was lower than their respective CA, as previously described (4). GH deficiency leads to slow bone growth and maturity, owing to delayed BA. The treatment effect at different time points was independent of the gender amongst pre-pubescent PSIS children in this study. This differed from previous studies (12) in which pre-pubescent boys had a greater response to hGH treatment than pre-pubescent girls with GHD who were small for their gestational age. Deficiencies in other pituitary hormones may weaken the responses of males, and the low number of patients may have contributed to the discrepancies. The BMI SDS of pre-pubescent boys tended to be higher than those of females, though the differences were not statistically significant. The increase in BMI SDS may have adverse effects on height growth in shorter male children, consistent with previous reports (14).

In previous studies on congenital IGHD patients, the GV in the first year of hGH therapy was 8.92±2.99 cm for males and 8.17±3.15 cm for females (15). The GV in the first year of hGH treatment was significantly higher than that of GHD patients (p<0.001 for males and p=0.004 for females), whilst the CA values were similar (p=0.170 for males and p=0.272 for females). The greater responses to hGH treatment in PSIS patients may be due to the higher sensitivity to hGH and/or severity of GH deficiency of these patients (16). The GV of patients in the Female-Group, Male-Group 1, Male-Group 2 and Male-Group 3 in this study were higher in the first year compared to the second year, consistent with previous studies and confirming that GHD children show faster linear growth during the initial stages of GH therapy (17) in addition to other studies on congenital MPHD (4).

Although the mean CA and mean BA of Male-Group 3 were both higher than those of the other two male groups, the GV during the first two years of hGH treatment was similar, which may be explained by the induction of puberty. The induction of puberty leads to a growth spurt which may explain the response to hGH treatment observed.

In previous GHD studies, only one third of patients reached normal adult height in response to hGH treatment (15). The duration of hGH treatment, age at initiation of hGH treatment and ethnicity may contribute to the discrepancies between this and previous studies. Although girls and boys had similar responses to hGH at different time points pre-puberty, females achieved better adult height than males in this study. The younger age at the initiation of hGH treatment of females and small sample size may explain this enhanced treatment effect.

A positive correlation between total height gain and GV in the first year of hGH treatment was in accordance with previous studies (18). The total height gain in the first year of hGH is an effective predictor of future height outcomes (19,20,21,22). Previous studies on GHD also indicate the importance of early treatment with hGH for IGHD patients (23,24). The negative relationship of BA and total height gain in this study also reflects a similar phenomenon in patients with PSIS and the total height gain may be greater if patients receive hGH at an earlier stage. It has been suggested that this is because early initiation of hGH permits a longer duration of treatment and larger gains in height (24) although height gains in the second year of therapy with hGH are consistently less impressive than those achieved in the first year of therapy.

### Study Limitations

The uneven number of male and female patients and differences in duration of hGH treatment may have influenced the results. Of particular note there were only four females in this study who attained adult height. It is not possible to reach a definite conclusion about the effect of hGH in female PSIS patients and the number of female patients is too low to compare with male patients reliably In addition data regarding the sexual development of PSIS patients was not collected and so analysis of the effect of hGH and gonadotropin therapy on pubertal development in PSIS patients was not performed. These limitations should be addressed in future studies.

## Conclusion

Males and females with MPHD caused by PSIS had a similar GV during hGH treatment before puberty. The GV during the first year of hGH treatment can predict future height outcomes for patients with MPHD caused by PSIS. PSIS patients may attain normal adult heights following hGH treatment.

## Figures and Tables

**Table 1:**
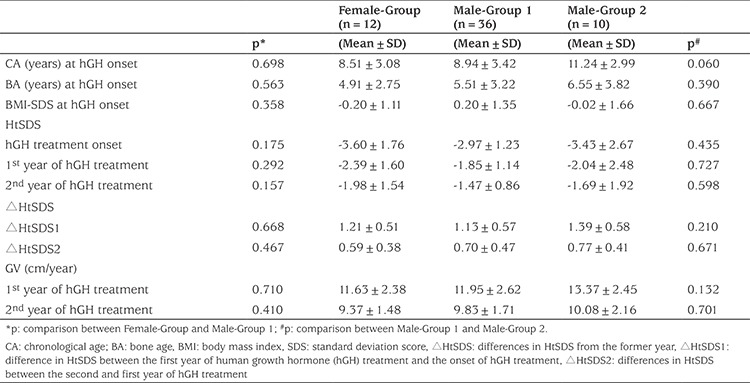
Characteristics of patients with pituitary stalk interruption syndrome treated with human growth hormone

**Table 2:**
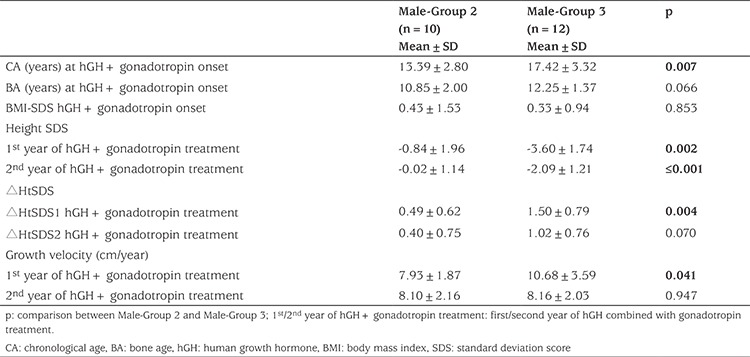
Characteristics of male patients with pituitary stalk interruption syndrome treated with both human growth hormone and gonadotropin
